# TRK inhibitors block NFKB and induce NRF2 in TRK fusion-positive colon cancer

**DOI:** 10.7150/jca.60845

**Published:** 2021-08-28

**Authors:** Sung-Hwa Sohn, Hee Jung Sul, Bohyun Kim, Bum Jun Kim, Hyeong Su Kim, Dae Young Zang

**Affiliations:** 1 Hallym Translational Research Institute, Hallym University Sacred Heart Hospital, Anyang, 14066, Republic of Korea.; 2 Department of Internal Medicine, Hallym University Medical Center, Hallym University College of Medicine, Anyang-si, Gyeonggi-do 14068, Republic of Korea.

**Keywords:** TPM3-NTRK1, TRK inhibitors, NRF2, NFκB

## Abstract

Tropomyosin receptor kinase (TRK) fusion is one of the oncogenic driver causes of colon cancer, and tropomyosin 3-neurotrophic receptor tyrosine kinase 1 (TPM3-NTRK1) fusion has been detected in the KM12SM cell line. In the present study, we investigated anticancer mechanisms in the KM12SM cell line using three different form of dovitinib (dovitinib (free base), dovitinib lactate (mono acid), and dovitinib dilactic acid (diacid)) and four TRK inhibitors (LOXO-101, entrectinib, regorafenib, and crizotinib). Exposure of TRK inhibitors at concentrations of 10 nM resulted in the apoptosis of KM12SM cells, whereas regorafenib had no effect. Treatment with all inhibitors except regorafenib also significantly increased the expression levels of the genes nuclear factor-erythroid 2-related factor 2 (NRF2) and glutamyl cysteine ligase catalytic subunit (GCLC) in KM12SM. These drugs significantly reduced expression of the phosphorylated proteins NFκB and COX-2 in the KM12SM cell line, and significantly attenuated KM12SM cell migration, according to a Transwell migration assay. Together, these results suggest that TRK inhibitors block products of carcinogenesis by negatively regulating the NFκB signaling pathway and positively regulating the antioxidant NRF2 signaling pathway.

## Introduction

Colorectal cancer can be caused by genetic mutations. Despite improved colonoscopy techniques, only 38% of colorectal carcinomas are detected at an early stage 1. Gene rearrangements, the most type of common molecular alteration, confer oncogenic properties to genes. Therefore, the treatment approach for colorectal cancer patients should carefully consider the molecular profile of the tumor 2.

The tropomyosin receptor kinase (TRK) family (TRK A-C) are major oncogenic drivers of various cancers, such as colorectal cancer, thyroid cancer, glioblastoma, cholangiocarcinoma, and lung cancer 3-5. TRK mutations are mutually exclusive with other oncogenic drivers, which makes TRK-positive cancer a distinct disease entity, similar to other oncogene-addicted cancers such as c-MET and ALK 6. Multi-kinase agents have activity against TRK A-C, but differ in their activity against other kinases. Many chemo preventive mechanisms have been shown to induce apoptosis and detoxification enzymes, including antioxidative enzymes, and inhibit inflammation and tissue invasion 7-10.

Nuclear factor erythroid 2-related factor 2 (NRF2) regulates the expression of antioxidative enzymes such as glutamyl cysteine ligase catalytic subunit (GCLC) 11, which plays multiple roles against oxidative stress. NRF2 also reduces the expression of inflammation factors such as cyclooxygenase-2 (COX-2) 12, 13, and previous studies have shown that the NFκB pathway is involved in inflammation and carcinogenesis. Therefore, the aim of this study was to determine whether a range of TRK inhibitors might exert anticancer effects via the NFκB/NRF2 pathway. Using apoptosis, gene, protein, and cell migration analyses and three different form of dovitinib (dovitinib (free base), dovitinib lactate (mono acid), and dovitinib dilactic acid (diacid)) and four TRK inhibitors (LOXO-101, entrectinib, regorafenib, and crizotinib) TRK inhibitors, we tested this hypothesis in the KM12SM cell line through tropomyosin 3-neurotrophic receptor tyrosine kinase 1 (TPM3-NTRK1) fusion.

## Methods

### Cell culture and reagents

Human colon cancer cell lines KM12SM, HT29, and HCT116 were obtained from the Korean Cell Line Bank (Seoul, Republic of Korea) and maintained in RPMI 1640 supplemented with 10% fetal bovine serum. The cells were cultured at 100% humidity and 5% CO_2_ at 37 °C. The NTRK inhibitors LOXO-101, entrectinib, dovitinib, dovitinib lactate, dovitinib dilactic acid, regorafenib, and crizotinib were purchased from Selleck Chemicals (Houston, TX, USA). We used the annexin V-APC/propidium iodide (PI) apoptosis detection kit (Thermo Fisher Scientific, Rockford, IL, USA).

### Transwell migration assay

A Transwell migration assay was performed using a 24-well Transwell chamber (8.0-μM-diameter pores) inserted into 24-well plates. We added 1 × 10^4^ cells in 300 μL serum-free RPMI 1640 medium to the upper chamber. NTRK inhibitors were diluted in RPMI 1640 medium to a final concentration of 10 nM and added to the lower chamber of each well. After 24 h of incubation, the medium and unmigrated cells in the upper chamber were removed, and the lower sides of the membranes were fixed with methanol and stained with hematoxylin and eosin. Images were taken using bright field microscopy (200× magnification).

### Growth inhibition assays

We measured the 50% inhibitory concentrations (IC_50_) of the NTRK inhibitors in KM12SM, HT29, and HCT116 cells using a specific MTS assay. We tested NTRK inhibitor concentrations of 10, 1, 0.1, 0.05, 0.0025, 0.00125, 0.001, 0.0001, 0.00001, and 0.000001 µM for 48 h. On the day of the proliferation assay, the medium was removed, and 200 µL fresh medium was added to each well of a 96-well plate, followed by 20 µL MTS solution. The plates were incubated at 37 °C for 2 h in a humidified environment with 5% CO_2_. The absorbance was measured at 490 nm using a microplate reader (Synergy 2 Multi-Mode Microplate Reader; BioTek, Winooski, VT, USA). The IC_50_ was calculated using nonlinear regression analysis with GraphPad Prism 5 software (GraphPad Software Inc., San Diego, CA, USA).

### Apoptosis analysis

KM12SM, HT29, and HCT116 cells were seeded into 6-well plates at a density of 5 × 10^4^ cells/mL and then treated with 10 nM of each NTRK inhibitor. Cell death was determined using an annexin V-APC/PI apoptosis detection kit (Thermo Fisher Scientific, Waltham, MA, USA) with a CytoFLEX flow cytometer (Beckman Coulter, Brea, CA, USA). The percentages of intact and apoptotic cells were calculated using CytExpert software (version 2.0; Beckman Coulter).

### Quantitative reverse-transcription PCR (qRT-PCR) analysis

To quantify mRNA expression, total RNA from each sample was reverse-transcribed into cDNA using the High Capacity cDNA Reverse Transcription Kit (Applied Biosystems, Foster City, CA, USA). qRT-PCR was performed using Power SYBR Green PCR Master Mix and a LightCycler 96 instrument (Roche Applied Science, Indianapolis, IN, USA). The transcript levels of glyceraldehydes-3-phosphate dehydrogenase (GAPDH) were used for sample normalization. The primer sequences used were as follows: NTRK1 (TRKA; forward: 5'-AAACCAGTGGATCTGCCAAC-3', reverse: 5'-ACGTAGCCGAAGAAACCTCA-3'), COX-2 (forward: 5'-TGAGCATCTACGGTTTGCTG-3', reverse: 5'-AACTGCTCATCACCCCATTC-3'), NRF2 (forward: 5'-AAACCAGTGGATCTGCCAAC-3', reverse: 5'-ACGTAGCCGAAGAAACCTCA-3'), GCLC (forward: 5'-GTGGATGTGGACACCAGATG-3', reverse: 5'-GCGATAAACTCCCTCATCCA-3') and glyceraldehyde 3-phosphate dehydrogenase (GAPDH) (forward: 5'-TTCACCACCATGGAGAAGGC-3', reverse: 5'-GGCATGGACTGTGGTCATGA-3').

### Immunoblot analysis

Immunoblot analysis was conducted using standard procedures. Commercially available primary antibodies were directed against anti-TRK (1:1,000; sc7268; Santa Cruz Biotechnology, Santa Cruz, CA, USA), anti-phospho-NFkB p65 (1:1,000; sc372; Santa Cruz Biotechnology), anti-COX-2 (1:1,000; sc19999; Santa Cruz Biotechnology), anti-phospho-ERK (1:500; #9101; Cell Signaling Technology), anti-ERK (1:1000; sc514302; Santa Cruz Biotechnology) and anti-GAPDH (1:4,000; sc32233; Santa Cruz Biotechnology).

### Statistical analyses

All data were statistically analyzed using GraphPad Prism 5 software (GraphPad Software). All values are presented as mean ± standard error of the mean (SEM). Statistical significance was determined using one-way analysis of variance (ANOVA), with *P* < 0.05 considered significant.

## Results

### Effects of NTRK inhibitors on colon cancer cells with and without NTRK gene fusion

**Figure 1:**
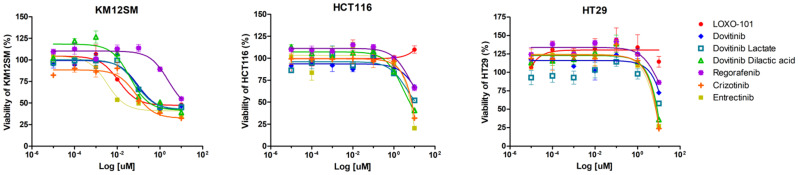
Effects of neurotrophic receptor tyrosine kinase (NTRK) inhibitors on colon cancer cells with or without NTRK gene fusion. KM12SM, HCT116, and HT29 cells were treated with various concentrations of NTRK inhibitors for 48 h.

We observed dose-dependent inhibitory effects of NTRK inhibitors in KM12SM (TPM3-NTRK1 fusion), HCT116 (NTRK3, p.I837T), and HT29 cells (**Figure 1**). The cells were treated with different concentrations of NTRK inhibitors for 48 h, and the optimal dose was determined by evaluating cell viability using an MTS assay. NRTK inhibitors inhibited cell viability in KM12SM cells, whereas NRTK inhibitors seldom had an effect on HCT116 and HT29 cells.

### Effects of NTRK inhibitors on cell apoptosis

**Figure 2:**
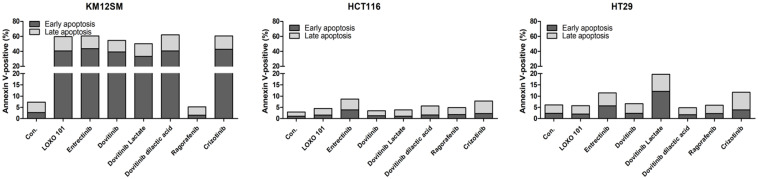
Apoptosis activity of NTRK inhibitors in colon cancer cells. Flow cytometric assay results for colon cancer cells treated with 10 nM of each NTRK inhibitor for 48 h.

To evaluate the effects of NTRK inhibitors on cell death in KM12SM, HCT116, and HT29 cells, we examined apoptosis by staining with annexin V-APC/PI, followed by flow cytometry (**Figure 2**), to assess early apoptotic and late apoptotic cell populations. Among the cell types, the death rate was best for KM12SM cells with LOXO-101, entrectinib, dovitinib, dovitinib lactate, dovitinib dilactic acid, cabozantinib, and crizotinib treatment. Apoptosis was seldom observed in HCT116 and HT29 cells (**Figure 2**). The addition of 10 nM regorafenib to KM12SM cells for 48 h had no significant effect on cell death.

### Anticancer effects of NTRK inhibitors in KM12SM cells

#### NTRK inhibitors activated the NRF2 signaling pathway in KM12SM cells

**Figure 3:**
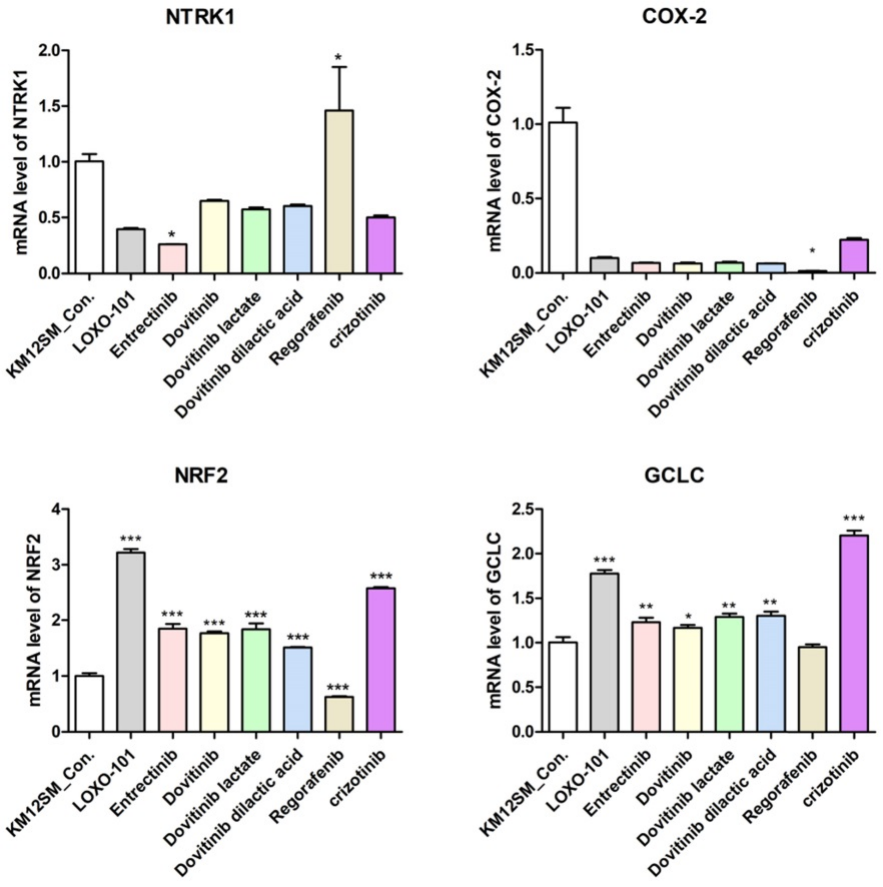
mRNA expression levels of NTRK1, cyclooxygenase-2 (COX-2), nuclear factor-erythroid 2-related factor 2 (NRF2) and NRF2-related anti-oxidant enzymes in colon cancer cell lines. NTRK1, COX-2, NRF2, and glutamyl cysteine ligase catalytic subunit (GCLC) mRNA expression levels were determined by quantitative reverse-transcription polymerase chain reaction (qRT-PCR). *p < 0.05; **p < 0.01; ***p <0.001.

We performed qRT-PCR to determine whether NTRK inhibitors activate the NRF2 signaling pathway for TPM3-NTRK1 fusion colon cancer in KM12SM cells (**Figure 3**). NTRK1 and COX-2 genes were downregulated when KM12SM cells were treated with 1 μM of all NTRK inhibitors except regorafenib, whereas NRF2 and GCLC genes were upregulated. KM12SM cells treated with regorafenib showed downregulation only of COX-2.

### NTRK inhibitors suppressed NFκB signaling pathway activity in KM12SM cells

**Figure 4:**
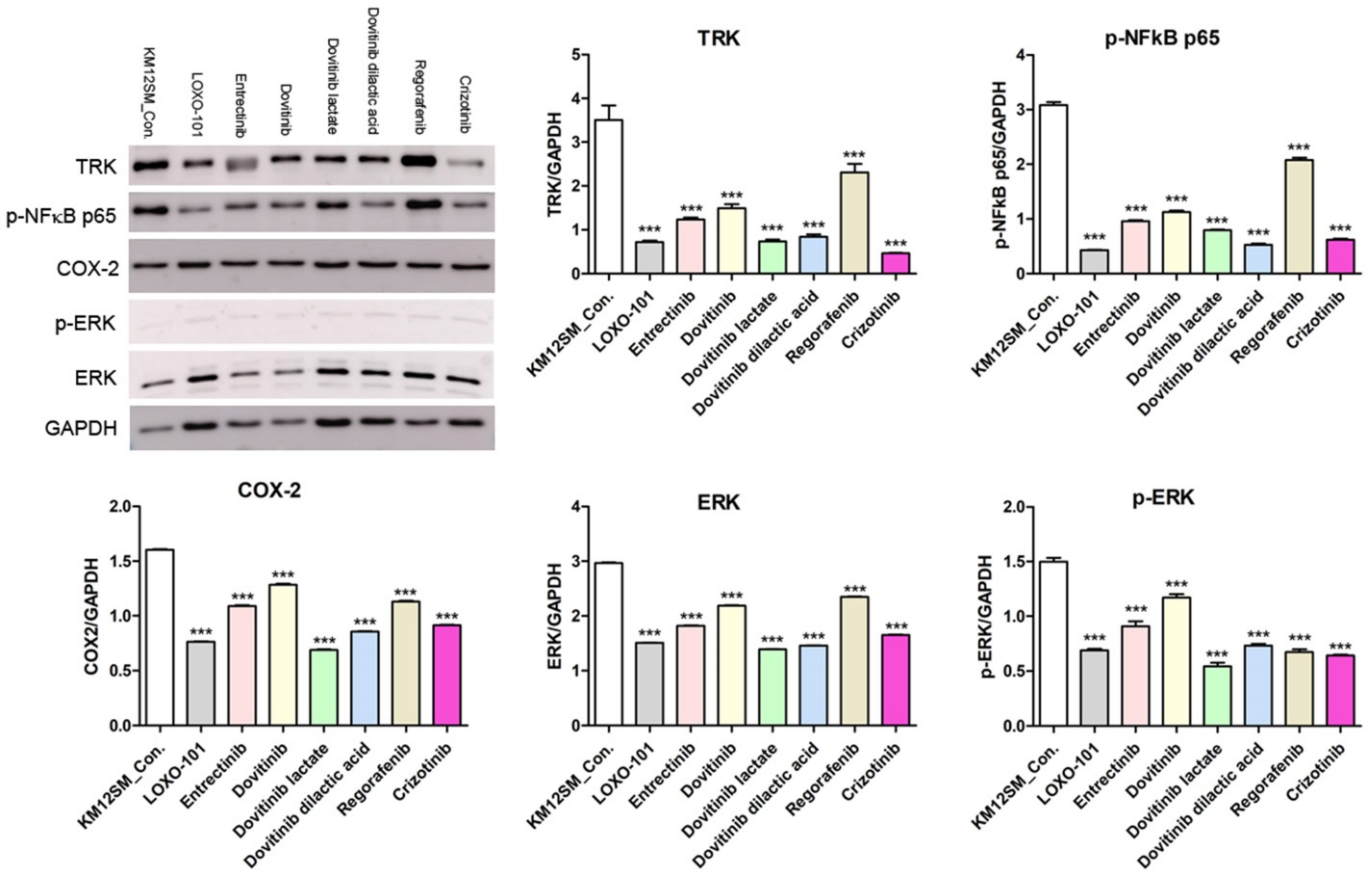
Protein expression levels of TRK, p-NFkB p65, COX-2, ERK and p-ERK in KM12SM cell lines. Protein expression levels of TRK, p-NFkB p65, and COX2 were determined using Western blot analysis. ***p < 0.001.

To further investigate the mechanisms of action of NTRK inhibitors in KM12SM cells, we analyzed phosphorylated NFκB (phospho-NFκB), COX-2, ERK, and phosphorylated ERK (phospho-ERK) proteins (**Figure 4**). Western blotting showed downregulation of phospho-NFκB, COX-2, ERK, and phospho-ERK in NTRK inhibitor-treated KM12SM cells.

### NTRK inhibitors attenuated migration in KM12SM cells

**Figure 5:**
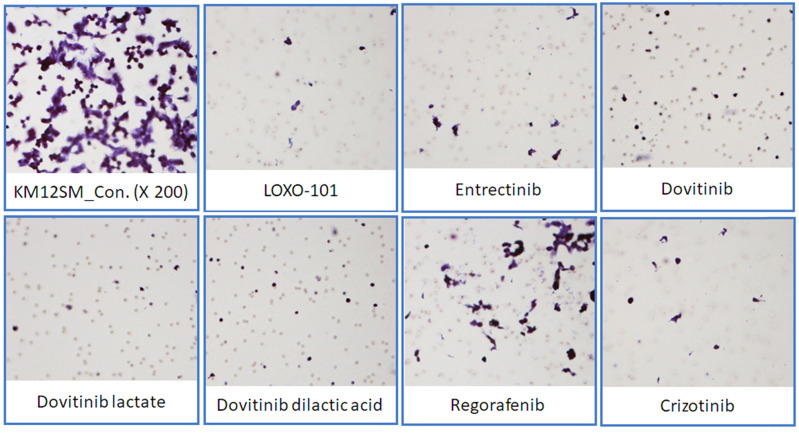
Transwell migration assay. Representative microscopic images of cells that migrated through the Transwell during the migration assay (hematoxylin and eosin staining, 200× magnification).

To determine the effects of NTRK inhibitors on KM12SM cell migration, we performed a Transwell migration assay (**Figure 5**). KM12SM cell migration was significantly decreased by NTRK inhibitor treatment compared with non-treated control cells.

## Discussion

The major finding of the present study was that six low-dose (10 nM) TRK inhibitors (LOXO-101, entrectinib, dovitinib, dovitinib lactate, dovitinib dilactic acid, and crizotinib) can induce apoptosis and attenuate cell migration in KM12SM cells harboring TPM3-NTRK1 fusion by regulating the NFκB and NRF2 signaling pathways. Low-dose (10 nM) regorafenib attenuated cell migration by regulating the NFκB signaling pathway in KM12SM cells, but did not induce apoptosis.

TPM3-NTRK1 was originally identified in cases of colorectal cancer 14, 15. TRK gene fusion is a highly therapeutically actionable driver of tumor growth. Several tumors are associated with gain-of-function mutations of genes coding for both serine/threonine and tyrosine kinases 16. TRK gene fusion is also characterized by ligand independent kinase activity and can occur in humans in a mutually exclusive manner with respect to other oncogenic drivers 3, 17. Although numerous 5' gene fusion partners have been identified, all studied variants appear to respond to targeted therapy 18, 19. In this study, we aimed to identify effective TRK inhibitors and determine whether they have anticancer effects via activation of the NFκB and NRF2 pathways in KM12SM cells with TPM3-NTRK1 fusion.

**Figure 6:**
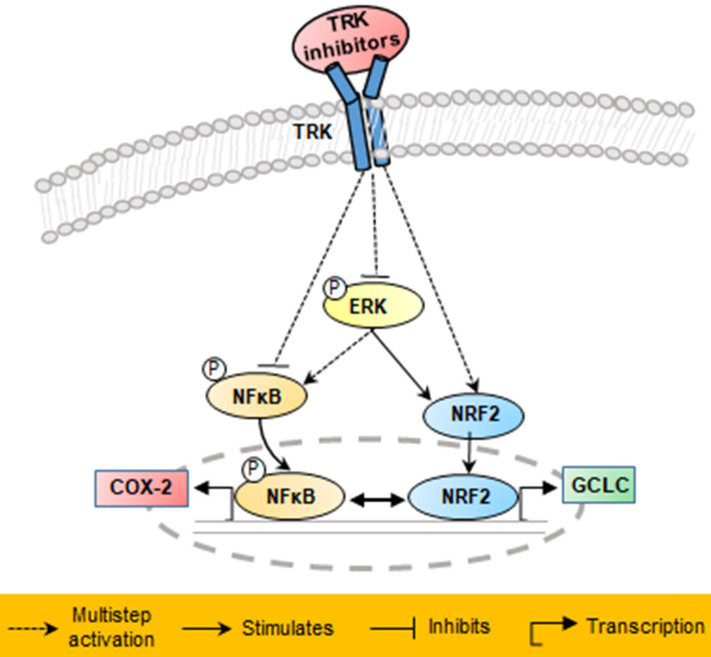
Schematic of proposed mechanisms involved in the anti-cancer effects in KM12SM cells of NTRK inhibitors.

We found that three different form of dovitinib and four TRK inhibitors from a TRK inhibitor screening library, i.e., dovitinib, dovitinib lactate, dovitinib dilactic acid, LOXO-101, entrectinib, regorafenib, and crizotinib, all reduced the viability of KM12SM cells *in vitro*; therefore, these compounds were selected for the present study. LOXO-101 had no effect on the HCT116 and HT29 cell lines, which lack TRK fusion, whereas a low dose (10 nM) of the other drugs affected all three cell lines, with and without TRK fusion (**Figure 1**). Apoptosis in KM12SM cells was highly induced (> 50%) by a low dose (10 nM) of each TRK inhibitor, except regorafenib, whereas these drugs had very little effect on HCT116 and HT29 cells (**Figure 2**). The balance between cell division and apoptosis depends on both oxidative stress and the mutation of important signaling molecules 11, 20. NRF2 is an antioxidant/detoxification enzyme that reduces oxidative injury and positively regulates the transcriptional activation of GCLC genes 21, 22. NRF2 also confers protection against carcinogenesis 23. However, a previous study reported that NRF2 was resistant to activation by chemotherapeutic agents 24. NRF2 is also activated by protein kinase signaling, which involves Akt and extracellular-related kinase (ERK) 25, 26. These Akt and ERK kinases signals may be required for subsequent NRF2-dependent transcription in the context of resistance to chemotherapeutic agents. In this study, low-dose drug delivery may have been associated with NRF2 signaling, which induces the expression of GCLC antioxidant genes; these drugs also suppressed COX-2, ERK, and phospho-ERK enzyme activity (**Figures 3, 4 and 6**). All of the TRK inhibitors attenuated NFκB signaling (**Figure 4 and 6**). Our Transwell migration assay revealed that all of the tested TRK inhibitors suppressed KM12SM cell migration (**Figure 5**).

In conclusion, the results of this study demonstrated that in KM12SM cells with TPM3-NTRK1 fusion, TRK inhibitors that induce NRF2 signaling were effective in colon cancer treatment at low concentrations, whereas those that did not induce NRF2 signaling were effective at high concentrations.

## References

[1] Jemal A, Murray T, Ward E, Samuels A, Tiwari RC, Ghafoor A, Feuer EJ, Thun MJ (2005). Cancer statistics, 2005. CA Cancer J Clin.

[2] Modest DP, Pant S, Sartore-Bianchi A (2019). 2019. Treatment sequencing in metastatic colorectal cancer. Eur J Cancer.

[3] Vaishnavi A, Capelletti M, Le AT, Kako S, Butaney M, Ercan D, Mahale S, Davies KD, Aisner DL, Pilling AB (2013). Oncogenic and drug-sensitive NTRK1 rearrangements in lung cancer. Nature medicine.

[4] Doebele RC, Davis LE, Vaishnavi A, Le AT, Estrada-Bernal A, Keysar S, Jimeno A, Varella-Garcia M, Aisner DL, Li Y (2015). An Oncogenic NTRK Fusion in a Patient with Soft-Tissue Sarcoma with Response to the Tropomyosin-Related Kinase Inhibitor LOXO-101. Cancer Discov.

[5] Vaishnavi A, Le AT, Doebele RC (2015). TRKing down an old oncogene in a new era of targeted therapy. Cancer Discov.

[6] Ricciuti B, Brambilla M, Metro G, Baglivo S, Matocci R, Pirro M, Chiari R (2017). Targeting NTRK fusion in non-small cell lung cancer: rationale and clinical evidence. Med Oncol.

[7] Wattenberg LW (1985). Chemoprevention of cancer. Cancer Res.

[8] Manson MM (2003). Cancer prevention - the potential for diet to modulate molecular signalling. Trends Mol Med.

[9] Sun SY, Hail N Jr, Lotan R (2004). Apoptosis as a novel target for cancer chemoprevention. J Natl Cancer Inst.

[10] Hou Z, Lambert JD, Chin KV, Yang CS (2004). Effects of tea polyphenols on signal transduction pathways related to cancer chemoprevention. Mutat Res.

[11] Dalton TP, Dieter MZ, Yang Y, Shertzer HG, Nebert DW (2000). Knockout of the mouse glutamate cysteine ligase catalytic subunit (Gclc) gene: embryonic lethal when homozygous, and proposed model for moderate glutathione deficiency when heterozygous. Biochem Biophys Res Commun.

[12] Huang SS, Chiu CS, Chen HJ, Hou WC, Sheu MJ, Lin YC, Shie PH, Huang GJ (2011). Antinociceptive activities and the mechanisms of anti-inflammation of asiatic Acid in mice. Evid Based Complement Alternat Med.

[13] Qi Z, Ci X, Huang J, Liu Q, Yu Q, Zhou J, Deng X (2017). Asiatic acid enhances Nrf2 signaling to protect HepG2 cells from oxidative damage through Akt and ERK activation. Biomed Pharmacother.

[14] Pulciani S, Santos E, Lauver AV, Long LK, Aaronson SA, Barbacid M (1982). Oncogenes in solid human tumours. Nature.

[15] Martin-Zanca D, Hughes SH, Barbacid M (1986). A human oncogene formed by the fusion of truncated tropomyosin and protein tyrosine kinase sequences. Nature.

[16] Alberti L, Carniti C, Miranda C, Roccato E, Pierotti MA (2003). RET and NTRK1 proto-oncogenes in human diseases. J Cell Physiol.

[17] Tognon C, Garnett M, Kenward E, Kay R, Morrison K, Sorensen PH (2001). The chimeric protein tyrosine kinase ETV6-NTRK3 requires both Ras-Erk1/2 and PI3-kinase-Akt signaling for fibroblast transformation. Cancer Res.

[18] Shaw AT, Ou SH, Bang YJ, Camidge DR, Solomon BJ, Salgia R, Riely GJ, Varella-Garcia M, Shapiro GI, Costa DB (2014). Crizotinib in ROS1-rearranged non-small-cell lung cancer. N Engl J Med.

[19] Davies KD, Doebele RC (2013). Molecular pathways: ROS1 fusion proteins in cancer. Clinical cancer research: an official journal of the American Association for Cancer Research.

[20] Nebert DW, Roe AL, Dieter MZ, Solis WA, Yang Y, Dalton TP (2000). Role of the aromatic hydrocarbon receptor and [Ah] gene battery in the oxidative stress response, cell cycle control, and apoptosis. Biochemical pharmacology.

[21] Kobayashi M, Yamamoto M (2006). Nrf2-Keap1 regulation of cellular defense mechanisms against electrophiles and reactive oxygen species. Adv Enzyme Regul.

[22] Mani M, Khaghani S, Gol Mohammadi T, Zamani Z, Azadmanesh K, Meshkani R, Pasalar P, Mostafavi E (2013). Activation of Nrf2-Antioxidant Response Element Mediated Glutamate Cysteine Ligase Expression in Hepatoma Cell line by Homocysteine. Hepat Mon.

[23] Kobayashi M, Yamamoto M (2005). Molecular mechanisms activating the Nrf2-Keap1 pathway of antioxidant gene regulation. Antioxid Redox Signal.

[24] Wang XJ, Sun Z, Villeneuve NF, Zhang S, Zhao F, Li Y, Chen W, Yi X, Zheng W, Wondrak GT (2008). Nrf2 enhances resistance of cancer cells to chemotherapeutic drugs, the dark side of Nrf2. Carcinogenesis.

[25] Kim J, Cha YN, Surh YJ (2010). A protective role of nuclear factor-erythroid 2-related factor-2 (Nrf2) in inflammatory disorders. Mutat Res.

[26] Torres M, Forman HJ (2003). Redox signaling and the MAP kinase pathways. Biofactors.

